# Effect of the *luxI/R* gene on AHL-signaling molecules and QS regulatory mechanism in *Hafnia alvei* H4

**DOI:** 10.1186/s13568-019-0917-z

**Published:** 2019-12-05

**Authors:** Xue Li, Gongliang Zhang, Yaolei Zhu, Jingran Bi, Hongshun Hao, Hongman Hou

**Affiliations:** 1 School of Food Science and Technology, Dalian Polytechnic University, No.1, Qinggongyuan, Ganjingzi District, Dalian, 116034 China; 2 Liaoning Key Lab for Aquatic Processing Quality and Safety, No.1, Qinggongyuan, Ganjingzi District, Dalian, 116034 China

**Keywords:** AHLs, *luxI/luxR*, *Hafnia alvei*, Genome, Transcriptome

## Abstract

*Hafnia alvei* H4 is a bacterium subject to regulation by a *N*-acyl-l-homoserine lactone (AHL)-mediated quorum sensing system and is closely related to the corruption of instant sea cucumber. Studying the effect of *Hafnia alvei* H4 quorum sensing regulatory genes on AHLs is necessary for the quality and preservation of instant sea cucumber. In this study, the draft genome of *H. alvei* H4, which comprises a single chromosome of 4,687,151 bp, was sequenced and analyzed and the types of AHLs were analyzed employing thin-layer chromatography (TLC) and high resolution triple quadrupole liquid chromatography/mass spectrometry (LC/MS). Then the wild-type strain of *H. alvei* H4 and the *luxI/R* double mutant (Δ*luxIR*) were compared by transcriptome sequencing (RNA-seq). The results indicate that the incomplete genome sequence revealed the presence of one quorum-sensing (QS) gene set, designated as *lasI/expR*. Three major AHLs, *N*-hexanoyl-l-homoserine lactone (C6-HSL), *N*-butyryl-l-homoserine lactone (C4-HSL), and *N*-(3-oxo-octanoyl)-l-homoserine lactone (3-oxo-C8-HSL) were found, with C6-HSL being the most abundant. C6-HSL was not detected in the culture of the *luxI* mutant (Δ*luxI*) and higher levels of C4-HSL was found in the culture of the *luxR* mutant (Δ*luxR*), which suggested that the *luxR* gene may have a positive effect on C4-HSL production. It was also found that AHL and QS genes are closely related in the absence of *luxIR* double deletion. The results of this study can further elucidate at the genetic level that *luxI* and *luxR* genes are involved in the regulation of AHL.

## Introduction

It is widely accepted that bacteria (both planktonic and biofilm cells) communicate with each other by sensing and releasing signaling compounds in a process commonly known as quorum sensing (QS) (Miller and Bassler 2000). This process is composed of an *N*-acyl-1-homoserine lactone (AHL) synthase (a LuxI-type family protein) and an AHL reporter (a LuxR-type family transcription regulator) (Kumar et al. 2016). LuxI family proteins are AHL synthases that catalyze the acylation of *S*-adenosylmethionine (SAM) via an acyl carrier protein (acyl-ACP) or CoA-aryl/acyl moieties to produce AHLs (Parsek et al. 1999; Dong et al. 2017). In this reaction, SAM supplies the amino group and acyl-ACP provides the acyl group. These molecules are used by Gram-negative bacteria as common signaling molecules and are involved in the regulation of biofilm formation and other properties of the bacteria (Galloway et al. 2012; Guo et al. 2017). When the concentration of an AHL reaches a threshold as a result of an increase in bacterial cell density, members of the LuxR protein family, which consist of the AHL receptors, would bind to the AHL to regulate the expression of numerous genes involved in various processes, including bioluminescence, pigment and/or antibiotic production, and virulence (Waters and Bassler 2005). So far, more than 37 genera of gram-negative bacteria have been identified that are regulated by AHL signaling molecules (Kimura 2014).

*Hafnia alvei* is a Gram-negative, facultatively anaerobic, rod-shaped and motile bacterium of the *Enterobacteriaceae* family. It is also an opportunistic pathogen and a dominant psychrophile found in putrid food (Vivas et al. 2008). *Hafnia alvei* is also commonly found in abundance among the communities of the *N*-acylhomoserine lactone (AHL)-producing food spoilers (Bruhn et al. 2004; Pinto et al. 2007). In addition, *H. alvei* is considered to be the most common biological contaminant in vacuum-packed frozen meat (Bruhn et al. 2004). This bacterium is known to produce *N*-(3-oxohexanoyl) homoserine lactone (3-oxo-C6-HSL) (Viana et al. 2009). Although *H. alvei* plays an important role either in the decay of meat or other vacuum-packed foods, little research has been carried out to determine the specific interaction mechanisms between AHLs and *luxI/luxR* genes in the *H. alvei* community.

In this study, we first studied the relationship between the *luxI/R* gene and different AHLs produced by *H. alvei* H4, first by using TLC and LC/MS, and then by sequencing the draft genome of *H. alvei* H4 to characterize its QS system at the molecular level. Further, RNA-seq-based transcriptome analysis was employed to obtain important insights into the gene expression patterns and regulatory elements of *H. alvei* (Sorek and Cossart 2010). To our knowledge, this has been the first transcriptome-wide study conducted on *H. alvei* to shed further light on the molecular mechanism of AHL-mediated QS.

## Materials and methods

### Bacterial strains and culture conditions

The bacterial strains used in this study were wild-type *H. alvei* (the strain number is CCTCC AB 2019337 *Hafnia alvei* H4) and three mutants, one lacking the *luxI* gene (Δ*luxI*), one lacking the *luxR* gene (Δ*luxR*), and another one lacking both *luxI* and *luxR* genes (Δ*luxIR*). All the mutants mentioned above were constructed by the laboratory before (Hou et al. 2019). In addition, the mini-Tn*5* mutant of *Chromobacterium violaceum* (CV026) was also used. Wild-type *H. alvei* H4 was previously isolated in our laboratory and from instant sea cucumber. The mutants were also constructed in our laboratory. All bacterial strains were stored at − 80 °C. CV026 was provided by the Chinese Academy of Agricultural Sciences (Gelencsér et al. 2012a, b). They were routinely cultured in Luria–Bertani (LB) broth (10 g tryptone per liter, 5 g yeast extract, 5 g NaCl per liter) with pH adjustment of 7. Except where indicated, bacteria were grown at 30 °C with shaking at 150 rpm.

### AHL extraction and characterization

AHLs were extracted from the supernatants of different bacterial culturesas previously described (Ravn et al. 2001). The extracted AHLs were first identified by TLC as described by Hou et al. (2018), but with some modification. In brief, a reverse phase C18 TLC plate (Merck, Darmstadt, Germany) was cut into 10 × 7.5 cm strips, and the extracted AHLs and standard AHLs [C4-HSL (800 µg/mL), C6-HSL (50 µg/mL) and 3-O-C8-HSL (0.5 mg/mL)] obtained from Sigma-Aldrich (St. Louis, MI, USA) were spotted onto one end of the strip about 1 cm from the edge of the strip, and with a spacing of 1 cm between spots. The spots were allowed to dry and the strip was developed in methanol/water (60%/40%, v/v). After development, they were air-dried in a 30 °C incubator, and the dried strip was then put into a culture dish and overlaid with a thin layer of LB top agar containing CV026, using a 1:2 volume ratio of CV026 suspension to LB agar medium. After setting, the plate was incubated at 30 °C for overnight. The presence of AHL would result in the appearance of purple spots in the agar, indicative of AHL-induced production of violacein (Chen et al. 2013; Okutsu et al. 2015).

The concentration of the major AHLs extracted from the bacterial culture supernatants were then quantified by LC/MS, with reference to a standard curve constructed for each of the three standards (C4-HSL, C6-HSL and 3-O-C8-HSL).

The conditions used for LC/MS are shown in Table 1. The setting of each parameter was adapted from the method of Hou et al. (2017) with slight modification. Elution of the samples was achieved by a gradient consisting of solvent A (water) and solvent B (acetonitrile). The gradient commenced with 90% A for 2 min and rising to 95% A in 1 min, and then remained at 95% A for 2 min. This was followed by a rapid decrease to 85% A in 1 min and remained at 85% A for 9 min. After that, the gradient was further dropped to 70% A in 5 min, and then to 50% A in 5 min.

**Table 1:** LC and MS conditions used in AHL detection

LC condition	LC condition	ϕ4.6 × 150 mm, 5 µm (Agilent)
	Column temperature	30 °C
	Inject volume	6 μL
	Mobile phase	Water/acetonitrile
	Flow rate	1.0 mL/min
	Measurement time	35 min
MS condition	MS	5500 AB SCIEX
	Ionization mode	ESI+
	Ion source	Turbo spray
	Curtain gas (CUR)	20 psi
	Ionspray voltage (IS)	5000 V
	Temperature (TEM)	450 °C
	Ion source gas1 (GS1)	30 psi
	Ion source gas2 (GS2)	10 psi
	Collision gas (CAD)	Medium

*AHL* acyl homoserine lactone, *LC*–*MS* liquid chromatography–tandem mass spectrometry

For the MS/MS system, optimum quantitative ion pairs (m/z) were determined under Multiple Reaction Monitoring (MRM) mode. The MRM parameters are shown in Table 2.

**Table 2:** Selected product ion m/z values and MRM parameters used for AHL analysis

Signals	Molecular formula	Q1/Q3	DP (V)	EP (V)	CE (V)	CXP (V)	Retention time
C4-HSL	C_8_H_13_NO_3_	172.1/102.1	90.0	10.0	15.0	13.0	5.01
C6-HSL	C_10_H_17_NO_3_	200.1/102.1	90.0	10.0	15.0	13.0	19.9
3-O-C8-HSL	C_12_H_19_NO_4_	242.1/102.1	90.0	10.0	15.0	13.0	23.2

### Genome sequencing of wild type *H. alvei* H4

Total DNA of *H. alvei* H4 was extracted from the cell pellet using a Wizard^®^ Genomic DNA Purification Kit (Promega, USA). The purity, concentration and integrity of the DNA sample were then tested. Specifically, the purity of the DNA preparation was measured by NanoDrop2000 (Thermo, USA). DNA concentration was measured by picogreen (QuantiFluor^®^ dsDNA System E2670, Promega, USA), and DNA integrity was determined by 1% agarose gel electrophoresis (LONZA, Switzerland). For quality tested samples, DNA fragments of the required length were recovered, and then a linker for cluster preparation was added. Finally, the whole genome re-sequencing was completed by the Illumina Hiseq 4000 sequencing technology (Hiseq X Ten Reagent Kit v2.5, Illumina, USA) and an Illumina PE library (450 bp) was constructed. The bioinformatics part mainly usedthe BWA software (http://bio-bwa.sourceforge.net/) to compare the obtained reads with the reference genome sequence. The SNP (single nucleotide polymorphism) and small indel information was detected by VarScan software (http://varscan.sourceforge.net/), and the sites with lower sequencing depth and lower quality values were filtered out. Then the annotation information of SNP and indel was obtained using the Annovar software (http://annovar.openbioinformatics.org/en/latest/) and the gff information of the reference genomes, *Hafnia alvei* strain HUMV-5920 (https://www.ncbi.nlm.nih.gov/pubmed/27313299) and *Hafnia alvei* FB1 (https://www.ncbi.nlm.nih.gov/genome/?term=Hafnia+alvei+) for annotation. Finally, the gene family enrichment analysis of SNP-related genes was performed based on the interpro annotation information of the SNP-corresponding genes.

### Preparation of *H. alvei* H4 transcriptome samples

RNA samples were harvested from wild-type *H. alvei* H4 and its mutant Δ*luxIR*. The samples were designated as W12-1, W12-2, W12-3 for wild type and IR-1, IR-2, IR-3 for Δ*luxIR*. In brief, 100 mL LB medium was inoculated with freshly grown preculture of the wild type or Δ*luxIR* and then cultured for 12 h at 30 °C with shaking at 150 rpm. After 12 h, the cultures were dispensed into 50-mL centrifuge tubes, chilled on ice, and centrifuged at 8000×*g* for 10 min. The supernatant was discarded, and the cell pellet was directly frozen in liquid nitrogen and stored at − 80 °C until further use. A total of six independent biological samples were analyzed by RNA-seq analysis, representing three replicates for each of the two strains.

### RNA extraction and quality verification

RNA-seq libraries for wild-type *H. alvei* H4 and Δ*luxIR* were constructed from each three different preparations of RNA. Total RNA was extracted from the cells using an RNAprep pure Cell/Bacteria Kit (Tiangen biotech, Beijing, China) according to the manufacturer’s instructions. RNA degradation and contamination were monitored by electrophoresis using 1% agarose gel (Bio-Rad, CA, USA). The purity, concentration and integrity of the DNA sample were then tested. Specifically, the purity of the RNA was checked by spectrophotometry using a NanoPhotometer^®^ spectrophotometer (IMPLEN, CA, USA). The concentration of RNA was measured with a Qubit^®^ RNA Assay Kit in Qubit^®^ 2.0 Fluorometer (Life Technologies, CA, USA), whereas the integrity of the RNA was determined using an RNA Nano 6000 Assay Kit of the Bioanalyzer 2100 system (Agilent Technologies, CA, USA).

### Library construction and sequencing

A total amount of 3 µg RNA per sample was used as input material for the six qualified RNA samples. Sequencing libraries were generated using NEBNext^®^ Ultra™ Directional RNA Library Prep Kit for Illumina^®^ (NEB, USA) according to the manufacturer’s recommendations, and index codes were added to attribute sequences to each sample. Briefly, mRNA was purified from the total RNA using poly-T oligo-attached magnetic beads. For prokaryotic samples, rRNA was removed using a specialized kit. In order to select cDNA fragments of 150–200 bp in length, the library fragments were purified with AMPure XP system (Beckman Coulter, Beverly, USA), and 3 µL USER Enzyme (NEB, USA) was then used with size-selected and adaptor-ligated cDNA at 37 °C for 15 min followed by 5 min at 95 °C before PCR. PCR was performed with Phusion HighFidelity DNA polymerase (NEB, USA), Universal PCR primers and Index (X) Primer. At last, all samples were purified (AMPure XP system) and the quality of the library was assessed by the Agilent Bioanalyzer 2100 system (Agilent Technologies, CA, USA).

### Data analysis

Clean reads were obtained by removing reads containing adapter, reads containing ploy-N and low-quality reads from raw data. Both the building index of the reference genome and the alignment of clean reads to the reference genome were performed using Bowtie2-2.2.3. Differential expression analysis of the IR12-W12 experimental group was performed using the DESeq R package (1.18.0) (Love et al. 2014) and DEseq was used to estimate the variance-mean dependence in count data from high-throughput sequencing assays and test for differential expression based on a model using the negative binomial distribution (Langmead and Salzberg 2012). The resulting *P*-values were adjusted using the Benjamini and Hochberg’s approach for controlling the false discovery rate. Genes with an adjusted *P*-value < 0.05 found by DESeq were assigned as differentially expressed (Anders and Huber 2010). All data were analyzed on the online platform of Novomagic Cloud Platform (https://magic.novogene.com/public/customer/login).

### Accession number(s)

The incomplete draft genome sequence of the *H. alvei* H4 has been uploaded to the National Center for Biotechnology Information (NCBI) database under the accession number SDAR00000000. Raw and processed transcriptome data have been deposited at the Gene Expression Omnibus (GEO) database under the accession number GSE137815.

## Results

### Secretion of AHLs by *H. alvei* H4 wild type and mutants

In order to comprehensively analyze the AHLs produced by the QS system of *H. alvei* H4, the AHLs extracted from the cultures of wild type and three mutants (Δ*luxI*, Δ*luxR* and Δ*luxIR*) at different time points were first subjected to TLC analysis.

**Fig. 1:**
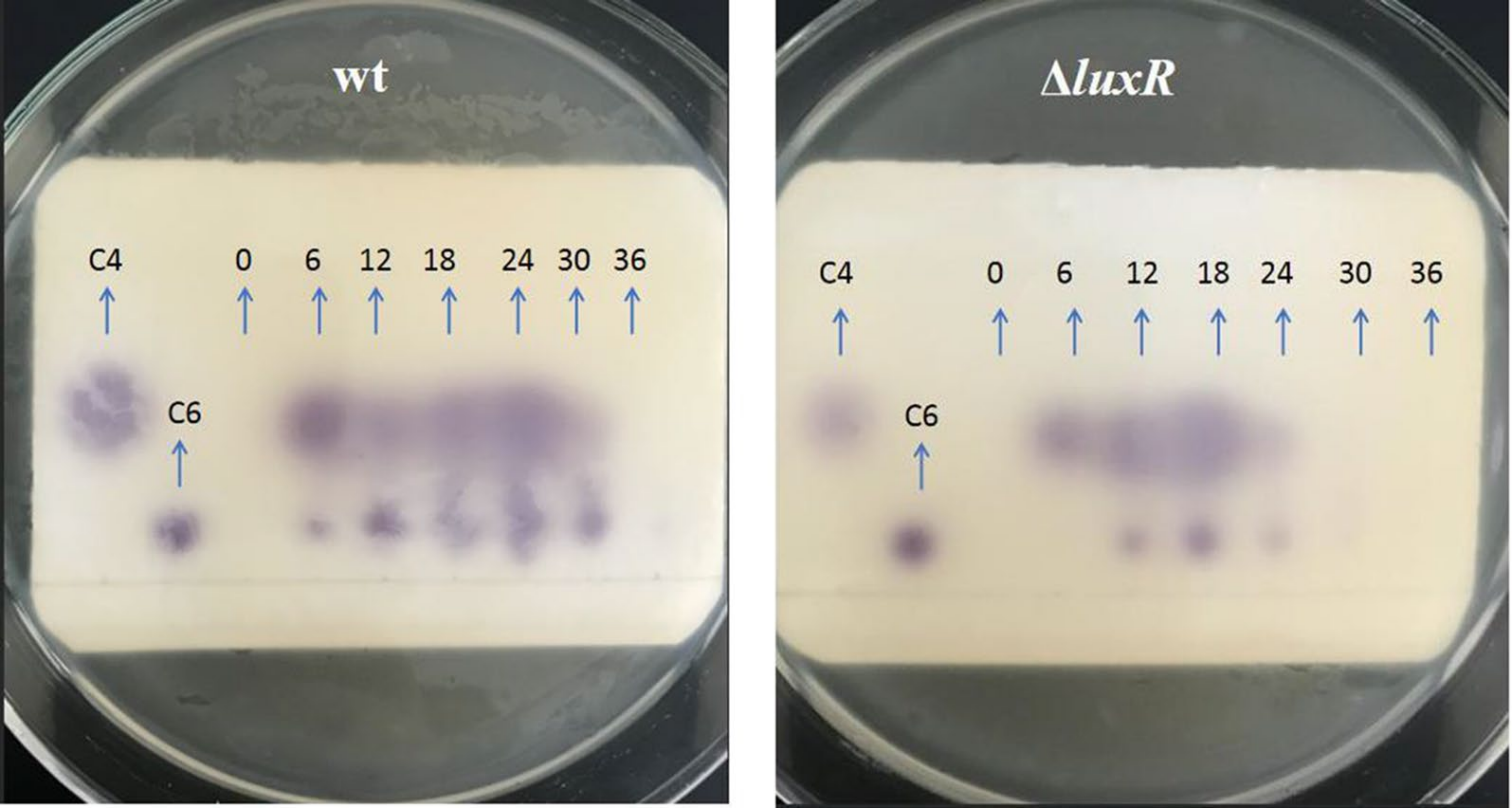
TLC bioassay of AHLs produced by *H. alvei* H4 wt and Δ*luxR* with *C. violaceum* CV026 as a sensor strain. Lane 1: C4-HSL standard AHL; Lane 2: C6-HSL standard AHL; Lane 3–Lane 9: 0–36 h every 6 h of AHL extracted from *H. alvei* H4 wild type and mutant strain culture supernatant

In the presence of the biosensor mini-Tn*5* mutant of *C. violaceum* (CV026), the AHL extracts from wild type and Δ*luxR* consistently produced two purple spots on the TLC plate, which corresponded to the two short-chain AHL standards, C4-HSL and C6-HSL (Fig. 1), whereas the extracts from Δ*luxI* and Δ*luxIR* did not produce any detectable AHL (data not shown) (Khajanchi et al. 2009). In the cases of wild type, changes in the production of C4-HSL and C6-HSL were followed by a similar pattern, and neither could be detected at zero time because the cell density was too low. Six hours after incubation, the two AHLs became detectable and their levels reached a peaked at 24 h and remained detectable even at 30 h, but no purple spot was detected at 36 h. Production of C4-HSL and C6-HSL were slightly different in Δ*luxR*, whereby no AHL was detected at 0 h. At 6 h, only C4-HSL was detected, and its level remained detectable until 24 h. For the C6-HSL, it was only detected at 12 h and no obvious purple halo could be seen after 24 h. Maximum levels of the two AHLs produced by Δ*luxR* occurred at 18 h, somewhat earlier compared with the wild type. Moreover, the maximum AHL level in the wild type and Δ*luxR* cultures corresponded to the maximum cell density (OD_600_ of 2.0 for wild type and 1.8 for Δ*luxR*).

**Fig. 2:**
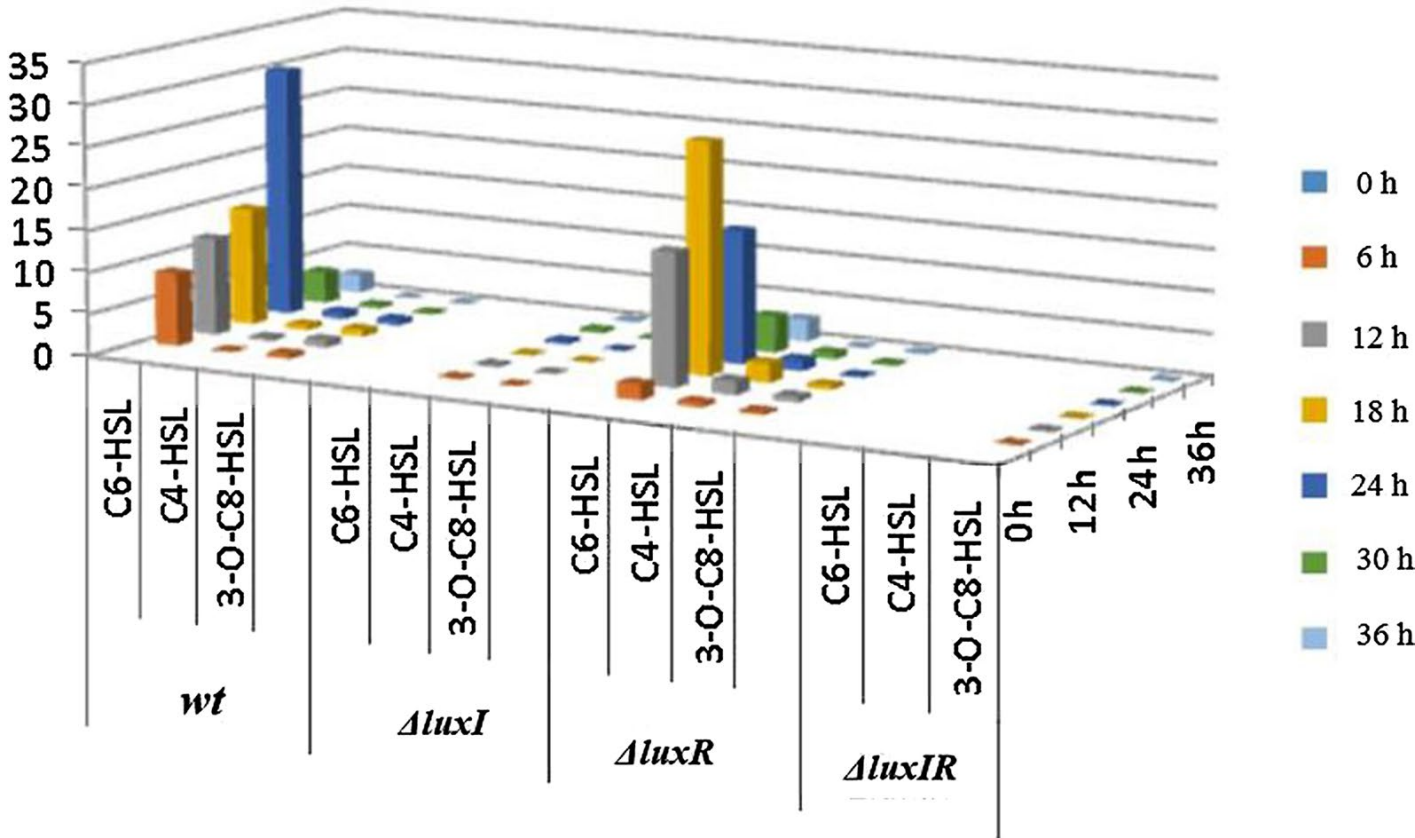
The AHL extracts of wild-type *H. alvei* H4 and its Δ*luxI,*Δ*luxR,*Δ*luxIR* mutants at different times were detected by LC/MS

The levels of AHLs produced by both wild type and Δ*luxR* were quantified by LC/MS. The result revealed the presence of three AHLs, C4-HSL, C6-HSL and 3-O-C8-HSL, with the first two present in much higher levels, peaking at 24 h in the case of wild type and at 18 h for Δ*luxR* (Fig. 2), consistent with the result obtained by TLC. Furthermore, in the case of Δ*luxR*, the concentration of C6-HSL did not change significantly over time, whereas the concentration of C4-HSL increased with incubation time. As for 3-O-C8-HSL, its concentration was extremely low, so no significant change was detected over time. Production of AHL by Δ*luxI* and Δ*luxIR* was quite different from wild type and Δ*luxR.* Not all the three AHLs produced by wild-type and Δ*luxR* were detected in the cultures of Δ*luxI* and Δ*luxIR*. For example, C6-HSL was completely undetectable in both Δ*luxI* and Δ*luxIR* cultures, and C4-HSL production in the Δ*luxI* culture was reduced but was not detected in Δ*luxIR* culture, whereas the production of 3-O-C8-HSL was significantly reduced in both cultures (Fig. 2). The result showed that at least, three distinct AHLs were produced by *H. alvei* H4, with C4-HSL being the main one, while 3-O-C8-HSL and C6-HSL being the minor AHLs. Furthermore, the result also demonstrated the importance of the *luxI* gene in the synthesis of C6-HSL and 3-O-C8-HSL, and suggested that the synthesis of C4-HSL might not depend on the *luxI* gene.

**Fig. 3:**
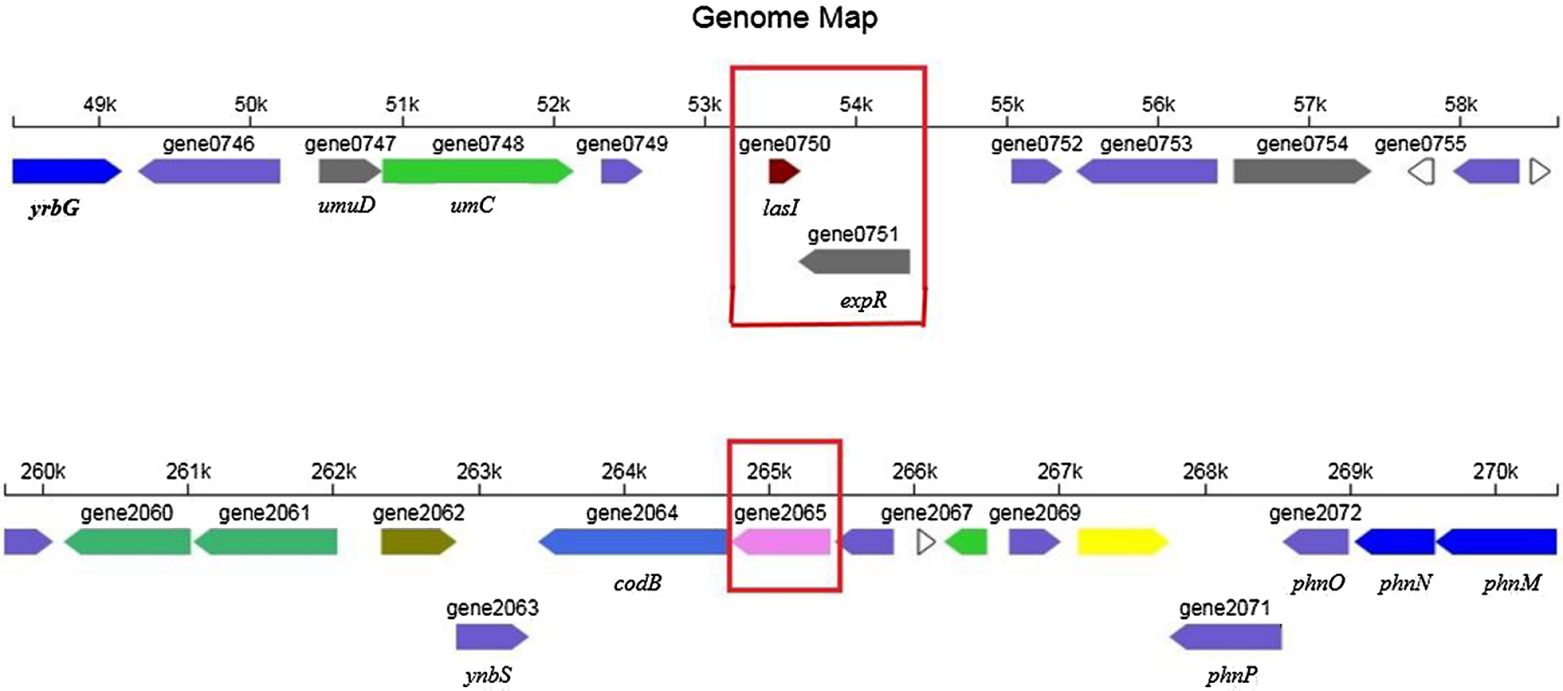
Arrows indicate the direction of transcription of the gene. The number below the gene arrow is the locus tag corresponding to the genome. Different colors represent the predictive function of different genes. *LuxI* family AHL synthase gene: *lasI* (gene0750), brown; *luxR* family transcription factor gene: *expR* (gene0751), gray; another *LuxR* family transcription factor gene “solo R”: gene2065, pink

In order to understand the molecular mechanisms of *luxI/luxR* gene and AHLs and further exploit its potentials, the genome of *H. alvei* H4 was sequenced and analyzed. The sequencing data revealed a high GC content (48.75%) in the genome of *H. alvei* H4, and 66 scaffolds were detected with a total genome length of 4,687,151 bp. To determine whether *H. alvei* H4 might contain an additional quorum sensing systems, its genome was searched for the presence of *luxI* and *luxR* homologous genes, and one *luxI*-type acyl-homoserine-lactone synthase gene (termed *lasI*, gene0750) and two *luxR*-type (termed *expR*, gene0751; *luxR* (unnamed), gene2065) transcriptional regulators were identified (Fig. 3). *expR* contained 19 bases overlapping with *lasI* and was located downstream of *lasI*, both of which were located in the second scaffold. In addition, another orphan *luxR*-type transcriptional regulator gene [solo R gene (Subramoni and Venturi 2009)] was found on the fourth scaffold without an adjacent *luxI* gene (Fig. 3). All these data were analyzed on the free online platform of Majorbio I-Sanger Cloud Platform (http://www.i-sanger.com). Additionally, due to the variability of gene names, *luxI/R* would still be used instead of the new annotated *lasI/expR*, which is consistent with the name previously used for this gene (Hou et al. 2017, 2018).

### Transcriptome analysis of AHL-related QS genes in *H. alvei* H4 wt and Δ*luxIR*

In order to elucidate the expression patterns of the *luxI/R* gene, transcriptome profiling was performed via RNA-sequencing (RNA-seq). Three independent biological replicates per strain were processed and sequenced. Read counts obtained for each sample were FPKM (fragments per kilobase of exon per million fragments mapped) normalized prior to being analyzed for differentially expressed genes. Subsequently, transcript reads were mapped to the *H. alvei* H4 genome. The mapped reads were filtered based on the mapping quality and only uniquely mapped reads were used for further analysis (Table 3). An extremely high correlation between each independent replicate and its respective counterpart be used, which indicated high reproducibility. Moreover, to investigate the changes in *H. alvei* H4 gene expression patterns following the knockout of the *luxIR* genes, the differentially expressed genes (DEGs) were aligned with the KEGG pathways to elucidate the biological functions of the DEGs. All DEGs were matched to 90 KEGG pathways (some genes could be simultaneously enriched into multiple pathways). The pathways that we focused on was not the most significantly enriched Bacterial chemotaxis (KEGG: ko02030) or other metabolic pathways, but QS pathway (KEGG: ko02024), and 5 down-regulated genes and 12 up-regulated genes were found to associate with this pathway.

**Table 3:** Statistics of reads that mapped to *Hafnia alvei* H4 genome per sample analysed

Sample	W12-1	W12-2	W12-3	IR-1	IR-2	IR-3
Total mapped reads (%)	98.73	98.7	98.88	98.8	98.81	98.82
Uniquely mapped reads (%)	97.81	97.84	98.02	97.95	98.09	98.16
RNA integrity number (RIN)	9.9	8.1	9.8	9.4	8.8	9.8

Samples W12-1, W12-2, W12-3 and IR-1, IR-2 and IR-3 represent biological replicates for RNA isolated from the H4 mutant at OD_600_ = 1.7

*OD* optical density, *IR LuxIR* gene mutant, *W* wild-type

## Discussion

In this study, three AHLs were quantified by TLC and LC/MS in order to investigate their secretion patterns and their relationship with the *luxI* and *luxR* genes. Firstly, stimulation or inhibition of violacein production by the AHL-dependent biosensor CV026 could be used in the TLC overlay to roughly detect the different AHL molecules that are structurally diverse (McClean et al. 1997). The sensitivity of CV026 to AHLs has enabled the different AHLs synthesized by *H. alvei* H4 to be separated and identified by TLC. The two short-chain AHLs (C4-HSL and C6-HSL) and one medium-chain AHL (3-O-C8-HSL) can stimulate the production of violacein by *C. violaceum* CV026 (Frederix and Downie 2011) and therefore, we concluded that AHLs were produced by the two tested *H. alvei* H4 strains (wild type and Δ*luxR*) because CV026 can only produce violacein in the presence of an exogenous supply of AHLs (AKabir et al. 2010). No obvious purple halo in response to the 3-O-C8-HSL standard was observed in the TLC result since CV026 is not sensitive to the 3-O-C8-HSL, and the level of 3-O-C8-HSL produced by *H. alvei* H4 may be low, and so *H. alvei* H4 could only weakly induce CV026 to produce violecein (data not shown).

AHL tends to accumulate when the cell density increases and once the concentration of AHL reaches a certain threshold, the whole bacterial population responds homogeneously upon the specific activation of target genes (Camilli and Bassler 2006). Therefore, it is not difficult to understand that at 0 h, no visible purple halo was observed because the number of bacterial cells in the culture was too low. Furthermore, due to the difference in color rendering properties of the different AHLs, the color reaction produced by CV026 showed a different purple halo size. The purple halo corresponding to C4-HSL observed in the TLC plate was relatively larger, but the LC/MS results did not indicate that abundant C4-HSL was synthesized by wild-type *H. alvei* H4. Among the three AHLs examined, C6-HSL was present in the lowest level, but it produced the largest spot in the TLC plate and also yielded the highest peak in the LC/MS spectrum. Therefore, the most abundant AHL produced by *H. alvei* H4 was C6-HSL. In addition, none of the three AHLs were detected in the Δ*luxI* and Δ*luxIR* cultures via TLC, which might be due to an inability to synthesize AHLs and hence, an impaired QS-regulated violacein production or the concentration of the AHL synthesized was too low to be detected, confirming that *luxI* could be the signaling synthase (Coutinho et al. 2013; Patzelt et al. 2013). Furthermore, these three AHLs were either not detected or detected in very low levels by LC/MS in the case of Δ*luxI,* suggesting that the *luxI* gene might control the synthesis of AHLs, and it was not surprising that AHLs could still be detected in the Δ*luxR* culture. Similarly, in *A. hydrophila*, mutation in the *ahyR* gene did not result in the loss of C4-HSL synthesis, and C4-HSL was present in the stationary phase of both the parent and *ahyR* mutant (Swift et al. 1999). Overall, we could initially conclude that the acyl homoserine lactone enzyme *luxI* in *H. alvei* H4 is important for the synthesis of AHLs, and that the receptor regulatory protein LuxR seems to be essential in this process.

To date, only three other *H. alvei* genomes have been sequenced, one is from strain ATCC 51873, isolated from the gut; another is BIDMC 31, which was investigated as part of the carbapenem resistance study, and was isolated from unspecified clinical source (http://www.ncbi.nlm.nih.gov/genome) (Tan et al. 2014). The third genome sequencing was performed for *H. alvei* FB1 isolated from fish meatballs, also a marine source, similar to *H. alvei* H4, which was isolated from instant sea cucumbers. Therefore, the strains that have been sequenced have certain reference significance for follow-up analysis. In this study, the genome of *H. alvei* H4 was sequenced and the positional relationship of QS-related genes was found throughout the genome data set. Firstly, in the *H. alvei* H4 genome, *luxI* and *luxR* were found to have opposite orientation and 19 base overlaps, with the same positional overlap as a pair of QS genes in *H. alvei* FB1 (Tan et al. 2014), whereas in *H. alvei* FB1, *luxI* is located downstream of *luxR*. Another similarity is that only one *luxI*-type gene was found in the *H. alvei* FB1 genome, except that it has six *luxR* genes, including a gene overlapping the *luxI* gene and five independent *luxR* genes. Studies have reported that conserved gene overlaps are another commonality observed in short, conserved topologies. Within the class of simple topologies, the majority of the cases are made up of the $${\vec{\text{R}}}\;{\vec{\text{I}}}$$ and $${\vec{\text{R}}}\;\overset{\lower0.5em\hbox{$\smash{\scriptscriptstyle\leftarrow}$}}{\text{I}}$$ the topologies that Goryachev termed type A and type B (Gelencsér et al. 2012a, b; Goryachev 2009, 2011). As for *H. alvei* H4, the positional relationship in the genome was found to belong to the second type of topology (Fig. 3). Such overlap is not uncommon in gene circuits where bacteria are tightly co-regulated (Krakauer 2000), such as restrictive modification systems (Kaw and Blumenthal 2010). The L1 type QS circuit of *P. aeruginosa* contains an overlap of 10 bp and the same overlap is 20 bp long in *P. fuscovaginae*. In contrast, *P. putida* has an L1 circuit where the R and L circuits are close (4 bp apart) but not overlapping (Gelencsér et al. 2012a, b). Other examples of the *luxR* gene have also been identified in locations that are separated from the QS circuit lacking the homologous N-AHL synthase, this *luxR* gene is referred to as solo (Hou et al. 2017) or orphan (Lequette et al. 2006) and is believed to allow the bacteria to perceive environmental stimuli and/or AHL produced by neighboring bacteria in that these *luxR* solos, which perceive environmental stimuli, contain the AHL-binding domain at the N terminus and a DNA-binding helix-turn-helix (HTH) domain at the C-terminus (Case et al. 2008; Cude and Buchan 2013; Fuqua 2006; González and Vittorio 2013; Tsai and Winans 2010). The specific function of most of these is currently unknown, however, among those *luxR* solos that have been studied up to now, many are interconnected with the resident AHL-QS systems. Or as previously found, the most striking feature of various circuit topologies is the potential negative regulation of *luxI*-*type* gene by *luxR*-type gene, in parallel with the well-known positive regulation. In other words, in many cases, *luxR* can both activate and suppress the *luxI* gene (Lequette et al. 2006). The phenomenon in the TLC experiment indicated a higher concentration of C4-HSL in the Δ*luxR* mutant compared with wt (Fig. 1), which is contrary to the secretion rule of C6-HSL. We suspected that this may be a negative regulation of *luxI* gene by *luxR* gene in the overlapping gene pair, resulting in the high expression of C4-HSL, a regulatory pattern parallel to the positive regulatory mode of C6-HSL. In the Δ*luxR*, the *luxR* gene with the overlapping fragment of the *luxI* gene was disrupted, and the inhibitory effect of *luxR* on *luxI* disappeared, thereby negatively regulating the expression of the *luxI* gene and expressing more C4-HSL. As gene overlaps and *luxR* solos have been reported in other bacteria, their existence in *H. alvei* H4 genome was not surprising. The different secretion patterns of C4-HSL, C6-HSL and 3-O-C8-HSL in wt, Δ*luxI*, Δ*luxR*, Δ*luxIR* suggested that the LuxR protein regulated AHL expression either positively or negatively.

**Fig. 4:**
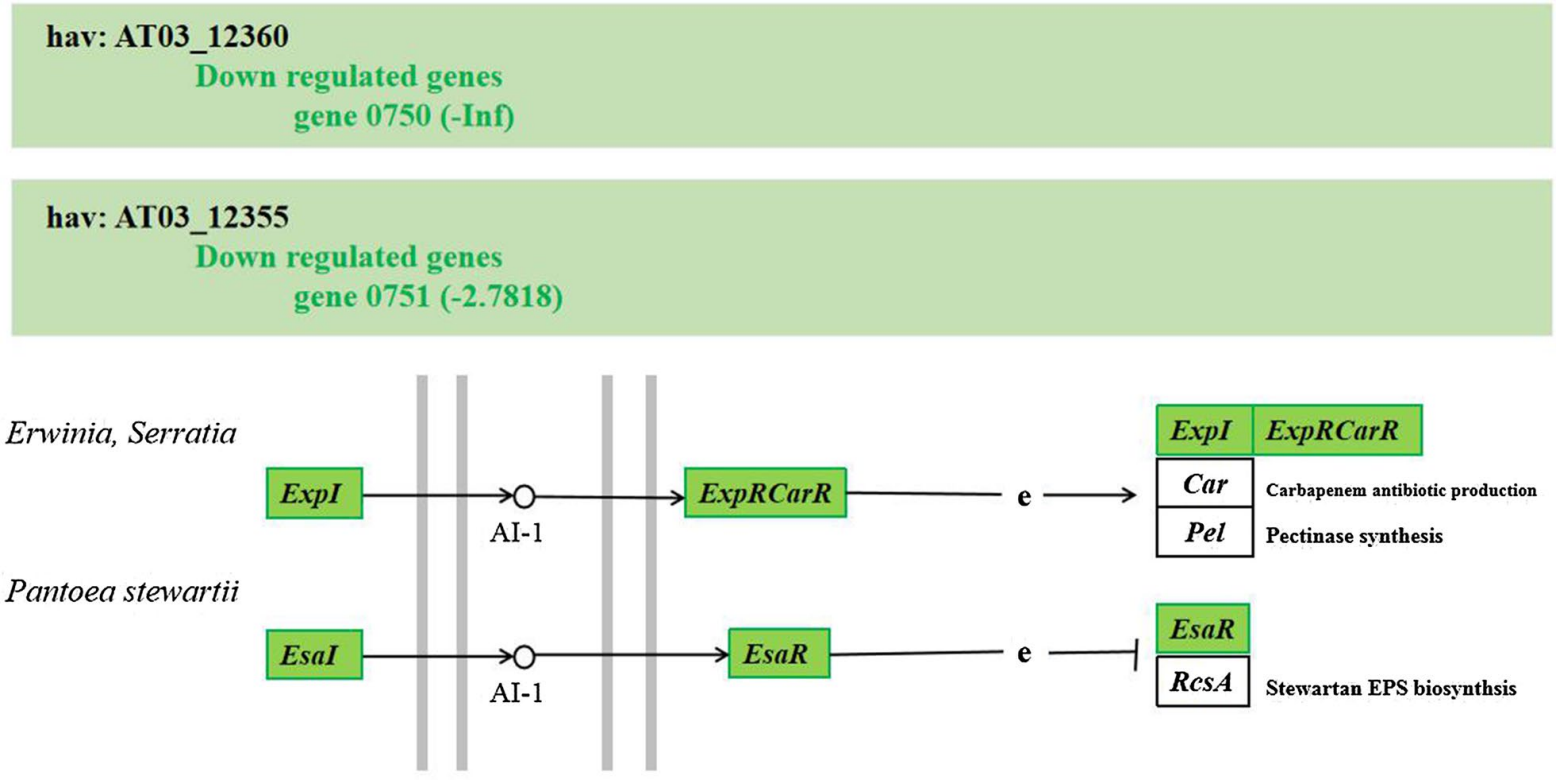
KEGG “quorum sensing” pathway based on RNA high-throughput sequencing analysis. Proteins appearing in the green boxes are down-regulated at the mRNA level

Although the genome is considered to be the blueprint for life, much information about the physiological or metabolic processes cannot be obtained directly from the genome (Wiegand et al. 2013). To this end, RNA-seq-based transcriptomics analysis was employed to provide a simpler and more efficient method (Nagalakshmi et al. 2010). For the selection of the subjects, we referred to the *mbaI/R*-type QS regulation model of methane-oxidizing bacteria. In methane-oxidizing bacteria, the addition of AHL extract to a strain containing the PmbaI-gfp plasmid, but not the mbaR-expressing plasmid, did not result in an increase in GFP fluorescence (Weeks et al. 2010). This process is, therefore, MbaR dependent and MbaR activates the expression of the synthase gene *mbaI*, upon signal binding, in a possible positive feedback loop. The combined results of TLC and LC/MS analysis in this study showed that the *luxR* gene may also act on the *luxI* gene, affecting the expression of C4-HSL. Therefore, in order to obtain more convincing evidence, we selected the *luxIR* double deletion strain and wild type to form a control group to further explore the changes in the transcription level of AHLs synthesis-related genes. The RNA sequencing results showed that > 800 genes were differentially expressed in *H. alvei* H4 when both *luxI* and *luxR* were deleted. These results indicated that *luxI/R* QS could have an immense influence on gene expression in *H. alvei* H4. For this section, based on the established DEGs and related KEGG pathways analyses, we focused on the QS response of genes controlling AHL synthesis in *H. alvei* H4 at the transcriptome level. Seventeen genes out of 59 genes fell into QS and RNA analysis of the “QS” pathway demonstrated that the key genes of the QS systems were down-regulated. In this pathway, either *ExpI/ExpR* in *Erwinia, Serratia* or *EsaI/EsaR* in *Pantoea stewartii* correspond to *lasI* (gene 0750)/*expR* (gene 0751) in the *H. alvei* genome, respectively. The gene0750 in *H. alvei* H4 was matched to the two *luxI*-type genes in the metabolic pathway map represented by “-Inf”, and “-Inf” that the readcount value of the gene was zero; the gene0751 in *H. alvei* H4 was matched to the two R genes indicated by “− 2.7818”, representing multiple down-regulation (Fig. 4). These indicated that after *luxIR* knockout, the gene controlling the synthesis of AHLs either does not express or is down-regulated, which is in line with the previously observed phenomenon that no AHL was detected in Δ*luxIR*.

Most studies devoted to the investigation of the relationship between AHL and QS genes at the transcriptome level have so far focused on several categories. In the first approach, studies have been performed to knock out some functional genes that control the biological phenotype and study the effects of DEGs on the other pathways and biological phenotypes (Khider et al. 2019; Wilf et al. 2013; Mori et al. 2018; Huang et al. 2019). The second approach is to add the corresponding AHL to the strain that has related knocked out genes (Puri et al. 2017) or the strain (Majerczyk et al. 2014) which does not produce AHL, and monitor the changes in the transcriptional regulation of DEGs before and after the addition of AHL. The third approach is to study the strain at different growth stages (or different cell densities) and this idea is widespread in most transcriptome sequencing. Our approach mainly combined genomic with transcriptome data. We focused on QS-related genes and QS pathways and explained the phenomena we observed in TLC and LC/MS at the molecular level. We have learned from the draft genome map that there could only be one QS system in *H. alvei* H4 and the special overlapping relationship of *luxI/R* gene pairs. This overlapping position of the *luxI/R* gene in the genome may have different mechanisms for the regulation of AHL. Furthermore, early sequencing efforts have demonstrated that individual genes can overlap or share one or more nucleotides with adjacent genes (Barrell et al. 1976; Sanger et al. 1977), and overlaps have been demonstrated to be potentially important in transcriptional and translational regulators as they might have influenced the evolution of genes (Keese and Gibbs 1992; Krakauer and Plotkin 2002). Therefore, the research in this paper might also provide some ideas for other genetic studies that contain the same special overlapping relationship. On the other hand, based on the results of TLC, LC/MS and the genomic data, we could only obtain one AHL-QS system from the draft genome, and there may be other QS systems in *H. alvei* H4, as in the case of *Pseudomonas chlororaphis* subsp. *aurantiaca* StFRB508 (StFRB508), two sets of AHL-synthase and AHL-receptor genes, *phzI/phzR* and *aurI/aurR* that we have identified in the incomplete draft genome of StFRB508, while the complete genome sequence revealed the presence of a third QS gene set, designated as *csaI/csaR (*Morohoshi et al. 2017). As for *H. alvei* H4, the presence of detectable AHL in LC/MS experiments conducted with Δ*luxI* and Δ*luxIR* could increase this possibility. QS signaling is very complex, but we have multiple verifications of its regulatory mechanism at the phenomenal and molecular levels, and it has broadened our appreciation of *H. alvei* H4 QS as a global regulatory system that affects many cellular functions. Moreover, transcriptome analysis has annotated multiple pathways other than QS, and we could better understand the other functions of *H. alvei* H4 through these pathways. In short, the specific mechanisms of the secretion of different AHLs and their association with the *luxI* and *luxR* genes and the transcriptional changes of differential genes in the different growth stages (or different cell densities) of bacteria and their functional characterization will be subjects of our future research, since more study is needed to determine the role of QS in this significant group of bacteria.

## Data Availability

The data of this research are inserted in the present article; other data is available if needed. The datasets supporting the conclusions of this article are available in the NCBI database in https://www.ncbi.nlm.nih.gov/nuccore/SDAR00000000.1/ and GEO database in https://www.ncbi.nlm.nih.gov/geo/query/acc.cgi?acc=GSE137815.

## Abbreviations

QS: quorum sensing
AHL: *N*-acyl-1-homoserine lactone
TLC: thin-layer chromatography
LC/MS: high resolution triple quadrupole liquid chromatography/mass spectrometry
SAM: *S*-adenosylmethionine
acyl-ACP: acyl carrier protein
C6-HSL: *N*-hexanoyl-l-homoserine lactone
C4-HSL: *N*-butyryl-l-homoserine lactone
3-oxo-C8-HSL: *N*-(3-oxo-octanoyl)-l-homoserine lactone
SNP: single nucleotide polymorphism
Solo R (orphans): QS-related LuxR AHL sensors/regulators which lack a cognate LuxI AHL synthase, which is unpaired QS LuxR-family proteins
